# Preferences for tobacco control health education among Chinese university students: A discrete choice experiment embedded in a randomized controlled trial

**DOI:** 10.18332/tid/220332

**Published:** 2026-07-01

**Authors:** Yu Chen, Jie Li, Xinjie Zhao, Guanmin Chen, Zhangyan Li, Ziliang Wang, Kin-Sun Chan, Xiaojing Guan

**Affiliations:** 1 School of Art and Communication, Fujian Polytechnic Normal University, Fuqing, China; 2 Chinese Center for Health Education, Beijing, China; 3 School of Journalism and Communication, Peking University, Beijing, China; 4 School of Cultural Industries Management, Communication University of China, Beijing, China; 5 School of Media and International Culture, Zhejiang University, Hangzhou, China; 6 School of Public Health, Peking University, Beijing, China; 7 Faculty of Social Sciences, University of Macau, Macau, China; 8 Hengqin In-Depth Cooperation HR Management Co., Ltd, Zhuhai, China

**Keywords:** discrete choice experiment, tobacco control, health education, university student

## Abstract

**INTRODUCTION:**

Tobacco use prevention among university students requires evidence on which educational program features students prefer. This study used a discrete choice experiment (DCE) embedded in a randomized controlled trial (RCT) to quantify Chinese never-tobacco-using university students’ preferences for attributes of a tobacco control health education program.

**METHODS:**

A DCE with six two-level attributes (organizer, content format, organization mode, frequency, educational content, delivery location) was administered to 260 never-tobacco-using university students enrolled in a Protection Motivation Theory (PMT)-based RCT. Seven choice sets, each containing two unlabeled alternatives, were presented via an online survey platform. Conditional logit models with cluster-robust standard errors estimated attribute preferences; Wald interaction tests assessed preference heterogeneity by trial arm, sex, parental smoking, and peer smoking.

**RESULTS:**

Of the 260 respondents, 192 (73.8%) were female, and 243 (93.5%) were undergraduates. Students significantly preferred video-based content (β= -0.1899; 95% CI: -0.2723 – -0.1074), online delivery (β= -0.1698; 95% CI: -0.2525 – -0.0872), and comprehensive cigarette-plus-e-cigarette content (β= -0.1320, 95% CI: -0.2147 – -0.0493). Content format (32.0%), delivery location (28.6%), and educational content (22.2%) together accounted for 82.8% of relative attribute importance. DCE preferences did not differ between trial arms (all Wald interaction p>0.33). A significant sex × delivery location interaction was observed (p=0.019).

**CONCLUSIONS:**

Chinese never-tobacco-using university students prefer video-based, online, comprehensive tobacco and e-cigarette education. These demand-side preferences may inform the design of future campus-based prevention programs. The absence of trial arm differences supports the validity of the embedded DCE–RCT design.

**CLINICAL TRIAL REGISTRATION:**

The study is registered on the official website of Chinese Clinical Trial Registry

**IDENTIFIER:**

ChiCTR2300068240

## INTRODUCTION

Tobacco use remains the leading preventable cause of death worldwide, responsible for more than 8 million deaths annually^1^. In China, which is home to approximately one-third of the world’s smokers, tobacco control has been identified as a national health priority under the Healthy China 2030 initiative^2,3^. University students constitute a particularly important target population for prevention efforts, as the transition to higher education represents a period of heightened vulnerability to tobacco initiation^4^. The 2021 national survey conducted by the Chinese Center for Disease Control and Prevention (China CDC), encompassing 124119 college students across 31 provinces, documented a current cigarette smoking rate of 7.8% and an e-cigarette ever-use rate of 10.1%, with the highest e-cigarette use observed among the age group of 15–24 years^4,5^. These figures underscore the dual tobacco threat – combustible and electronic – confronting Chinese university populations.

Tobacco control health education programs in Chinese universities vary considerably in their design, encompassing differences in who organizes and delivers the content, the format and modality of delivery, session frequency, the scope of educational content, and whether sessions are conducted online or in person^6-8^. A randomized controlled trial (RCT) grounded in Protection Motivation Theory (PMT) demonstrated that theory-driven, peer-delivered health education interventions can influence cognitive precursors to e-cigarette use among Chinese university students^9,10^. However, the effectiveness of such programs may depend not only on their theoretical basis but also on whether their delivery characteristics align with students’ preferences^11^.

Discrete choice experiments (DCEs) offer a well-established stated-preference methodology for quantifying the relative importance of product or service attributes^12-14^. Grounded in random utility theory^15,16^, DCEs present respondents with repeated choices between hypothetical alternatives characterized by systematically varied attribute levels, enabling estimation of the marginal utility associated with each attribute^14^. Unlike Likert-scale rating approaches, where all attributes may receive high ratings, DCEs force trade-offs between features, thereby revealing which attributes respondents truly prioritize. Since the seminal review by de Bekker-Grob et al.^12^, DCEs have been applied with increasing frequency in health economics and public health^13^. In the tobacco control domain, DCEs have been employed to investigate smoking cessation treatment preferences^17,18^, the design of financial incentive programs for quitting^19^, the effects of cigarette packaging on adolescent perceptions^20^, and cessation message framing^13^. Among university student populations specifically, Choi and Templin^17^ used a DCE to examine US college students’ preferences for tobacco treatment services. In China, DCEs have been applied to examine university students’ preferences for physical activity incentive programs^21^ and HPV vaccination services^22^, demonstrating the feasibility of this methodology with Chinese student populations. However, no published DCE has examined Chinese university students’ preferences for tobacco control health education program design.

The present study was embedded in a PMT-based online RCT that evaluated the preliminary effects of a peer-delivered health education intervention on tobacco-related cognitions among Chinese university students^9^. The embedding of a DCE within an RCT offers a distinctive methodological opportunity, it permits simultaneous collection of preference data and experimental evaluation of whether prior exposure to a health education intervention influences stated preferences, thereby providing an internal validity check for the embedded design^23,24^. To our knowledge, this approach has not previously been applied in the tobacco control health education context.

The objectives of this study were: 1) to estimate the relative importance of six tobacco control health education program attributes among Chinese university students who have never used tobacco; 2) to test whether DCE preferences differed between the intervention and control arms of the parent RCT, thereby assessing the validity of the embedded design; and 3) to explore preference heterogeneity by sex, parental smoking status, and peer smoking status.

## METHODS

### Study design and setting

This study employed a DCE embedded within a two-arm, single-blind, randomized controlled pilot trial that evaluated PMT-based peer-delivered tobacco control education among Chinese university students. The trial received ethical approval from the Ethics Committee of the Health Science Center, Peking University (IRB00001052-23001; approved in February 2023) and was prospectively registered with the Chinese Clinical Trial Registry (ChiCTR2300068240) on 11 February 2023. The study was conducted in accordance with the Declaration of Helsinki. All participants provided informed consent electronically before enrollment. The trial was conducted entirely online via the WenJuanXing platform between February and May 2023. The DCE module was integrated as part of the same online survey instrument administered at the 4-week follow-up assessment; participants first completed the follow-up questionnaire items and then proceeded to the DCE choice tasks within the same survey session. This study is reported in accordance with the DIRECT checklist for DCEs in health^25^ (Supplementary file).

The PMT-based intervention consisted of a 1-month peer education program in which 20 trained peer educators (15 females, 5 males, aged 18–24 years) delivered PMT-based messages through weekly one-on-one conversations via WeChat voice calls or messaging, approximately 10 minutes each. The intervention group received messages systematically addressing all seven PMT constructs across four weeks: Week 1 (perceived severity and vulnerability), Week 2 (intrinsic and extrinsic rewards), Week 3 (response efficacy and self-efficacy), and Week 4 (response costs). The control group received different messages about e-cigarette health risks (severity and vulnerability only) each week, ensuring equal contact time without the comprehensive PMT approach.

### Participants and recruitment

Eligible participants were full-time university students aged 18–24 years who self-reported never having used any tobacco product, including cigarettes and e-cigarettes. Recruitment was conducted from 12 to 28 February 2023 through online advertisements and digital platforms (WeChat). Students from 127 universities across mainland China were enrolled. Those who met the eligibility criteria and provided informed consent were randomized to either the intervention or control arm using simple randomization with concealed allocation: an independent statistician prepared 20 opaque envelopes, each containing 20 folded papers (10 marked ‘1’ for intervention, 10 marked ‘2’ for control); after baseline assessment, the research coordinator drew one paper from an envelope without replacement. Participants remained blinded to allocation throughout the study. Of the 289 participants who completed baseline assessment (144 intervention, 145 control), 260 (89.97%) provided valid DCE responses at the 4-week follow-up and were included in the present analysis, with 130 respondents in each arm.

Using the rule of thumb proposed by de Bekker-Grob et al.^27^, the minimum sample size for a DCE is calculated as n ≥ 500/(S×A) = 500/(7×2) = 36 per subgroup. Our sample of 260 substantially exceeds this minimum.

### Ethical approval

This study received ethical approval from the Ethics Committee of the Health Science Center, Peking University (IRB00001052-23001; approved February 2023). The study was conducted in accordance with the Declaration of Helsinki. All participants provided informed consent electronically before enrollment. The trial was registered prospectively in the Chinese Clinical Trial Registry (ChiCTR2300068240) on 11 February 2023.

### DCE design

Six attributes, each with two levels, were selected on the basis of a structured attribute development process. First, a literature review of tobacco control education programs in Chinese universities identified an initial list of 10 potential attributes. Second, expert consultation with five public health educators reduced this list to eight attributes based on relevance, policy modifiability, and plausibility of attribute levels. Third, three semi-structured focus group discussions with university students (6–8 participants each) further refined the list to the final six attributes by eliminating two attributes (session duration and incentive provision) that students considered either non-negotiable or irrelevant to their decision-making^23,24,28^. The attributes captured key dimensions of program design: organizer (teacher/expert vs student-led), content format (video vs text/image), organization mode (teaching-led vs self-study), frequency (more than once per semester vs once per semester), educational content (both cigarette and e-cigarette harms vs e-cigarette harms only), and delivery location (online vs offline/in-person). The final attribute and level specifications are presented in Table 1.

**Table 1:** DCE attributes, levels, and coding in a discrete choice experiment of tobacco control health education preferences among Chinese university students, February–May 2023 (N=260)

*Attribute*	*Level (+1)*	*Level (-1)*	*Variable*	*Coding*	*Reference*
**Organizer**	Teacher or expert	Student	Organizer	Effects (+1/-1)	-1
**Content format**	Text/image	Video	Format	Effects (+1/-1)	-1
**Organization mode**	Teaching-led	Self-study	Mode	Effects (+1/-1)	-1
**Frequency**	>1 per semester	1 per semester	Frequency	Effects (+1/-1)	-1
**Educational content**	E-cigarette harms only	Cigarette + e-cigarette harms	Content	Effects (+1/-1)	-1
**Delivery location**	Offline (in-person)	Online	Location	Effects (+1/-1)	-1

Effects coding assigns +1 to one level and -1 to the reference level. Negative coefficients indicate preference for the -1 coded (reference) level. DCE: discrete choice experiment.

The DCE questionnaire was pilot tested with two student focus groups (n=10 each, total 20 participants) who also contributed to the attribute development process. The pilot testing confirmed that the choice tasks were comprehensible, the attribute descriptions were clear, and the survey completion time was acceptable. No modifications to the choice set structure were made following the pilot.

A main-effects fractional factorial design was used to construct seven choice sets, each containing two unlabeled alternatives (Program A and Program B)^16,24^. Effects coding (+1/-1) was applied, with the reference level coded as -1 for each attribute. The maximum pairwise correlation among attribute columns was r= -0.17, confirming adequate near-orthogonality. All respondents completed all seven choice sets in a fixed order using a binary forced-choice format with no opt-out option.

### Statistical analysis

Baseline characteristics were summarized as frequencies and percentages for categorical variables, with comparisons between trial arms conducted using chi-squared tests.

Preferences were estimated using conditional logit (CL) models based on random utility theory^.15,16^ The utility function was specified as:

U = β1·Organizer + β2·Format + β3·Mode + β4·Frequency + β5·Content + β6·Location + ε

where positive coefficients indicate preference for the +1 coded level and negative coefficients indicate preference for the -1 coded (reference) level^26^. Relative attribute importance was calculated as the absolute value of each coefficient divided by the sum of all absolute coefficient values, expressed as a percentage^14,26^.

To validate the embedded design, separate CL models were fitted for the intervention arm (n=130) and the control arm (n=130), and Wald tests compared corresponding coefficients^26^. Exploratory subgroup analyses were conducted by sex, parental smoking status (neither parent vs at least one parent smokes), and peer smoking status (no close friends smoke vs at least one close friend smokes), with Wald interaction tests assessing heterogeneity. All analyses were performed using Python 3.10 with the statsmodels package (version 0.14), using maximum likelihood estimation with the BFGS optimization algorithm. Standard errors were computed using the sandwich (robust) variance estimator to account for within-respondent clustering. Statistical significance was set at p<0.05 (two-tailed).

## RESULTS

### Sample characteristics

A total of 260 never-tobacco-using university students provided valid DCE responses, generating 1820 choice observations across 7 choice sets. The sample was predominantly female (192/260; 73.8%) and enrolled in undergraduate programs (243/260; 93.5%). Among the respondents, 167 (64.2%) reported at least one parent who smokes, and 158 (60.8%) reported having close friends who smoke. The two trial arms were well balanced, with 130 respondents in each (Table 2). Chi-squared tests revealed no statistically significant differences in any baseline characteristic between arms (all p>0.05).

**Table 2:** Baseline characteristics of DCE respondents by trial arm in a discrete choice experiment embedded in an RCT of tobacco control health education, Chinese universities, February–May 2023 (N=260)

*Characteristics*	*Category*	*Total (N=260) n(%)*	*Intervention (N=130) n(%)*	*Control (N=130) n(%)*	*χ²*	*p*
**Sex**	Male	68 (26.2)	33 (25.4)	35 (26.9)	0.02	0.888
	Female	192 (73.8)	97 (74.6)	95 (73.1)		
**Degree**	Undergraduate	243 (93.5)	120 (92.3)	123 (94.6)	0.86	0.652
	Postgraduate	11 (4.2)	7 (5.4)	4 (3.1)		
	Other	6 (2.3)	3 (2.3)	3 (2.3)		
**Parental smoking**	Neither parent	93 (35.8)	49 (37.7)	44 (33.8)	0.27	0.605
	At least one parent	167 (64.2)	81 (62.3)	86 (66.2)		
**Peer smoking**	No close friends	102 (39.2)	47 (36.2)	55 (42.3)	0.79	0.374
	At least one friend	158 (60.8)	83 (63.8)	75 (57.7)		
**Trial arm**	Intervention	130 (50.0)	130			
	Control	130 (50.0)		130		

Comparisons between arms conducted using chi-squared tests. DCE: discrete choice experiment. RCT: randomized controlled trial.

### Intervention versus control arm comparison

To test whether prior exposure to the PMT-based health education intervention influenced DCE preference measurement, separate conditional logit models were estimated for the intervention arm (n=130) and the control arm (n=130). Wald interaction tests revealed no statistically significant differences in any attribute coefficient between arms (all p>0.33) (Table 3). The minimum interaction p-value was 0.326 for delivery location.

**Table 3:** Conditional logit results by trial arm in a DCE of tobacco control health education preferences, Chinese universities, February–May 2023 (intervention N=130; control N=130)

*Attribute*	*Int β*	*Int SE*	*Int p*	*Ctrl β*	*Ctrl SE*	*Ctrl p*	*Δβ*	*SE(Δ)*	*p _interaction*
**Organizer**	0.0712	0.0597	0.233	0.0014	0.0592	0.981	0.0697	0.0841	0.407
**Content format**	-0.2200	0.0600	<0.001	-0.1623	0.0592	0.006	-0.0577	0.0843	0.494
**Organization mode**	-0.0200	0.0597	0.738	0.0022	0.0591	0.970	-0.0222	0.0840	0.792
**Frequency**	0.0202	0.0602	0.737	0.0922	0.0591	0.119	-0.0720	0.0843	0.393
**Educational content**	-0.0921	0.0601	0.126	-0.1705	0.0594	0.004	0.0785	0.0845	0.353
**Delivery location**	-0.2122	0.0603	<0.001	-0.1292	0.0592	0.029	-0.0830	0.0845	0.326

Effects coding (+1/-1). Wald interaction test: z = Δβ/SE(Δ); two-tailed p-values. No interaction reached p<0.05. Int: intervention. Ctrl: control. DCE: discrete choice experiment.

### Full-sample preference estimates

The conditional logit model for the full sample (n=260; 1820 choice observations; log-likelihood= -1198.34) identified three attributes with statistically significant coefficients (Table 4). Content format had the largest absolute coefficient (β= -0.1899, SE=0.0421, 95% CI: -0.2723 – -0.1074, p<0.001), with the negative sign indicating preference for the -1 coded level (video). Delivery location was the second most influential attribute (β= -0.1698, SE=0.0422, 95% CI: -0.2525 – -0.0872, p<0.001). Educational content was the third significant attribute (β= -0.1320, SE=0.0422, 95% CI: –0.2147 – -0.0493, p=0.002).

**Table 4:** Conditional logit results, full sample, in a DCE of tobacco control health education preferences, Chinese universities, February–May 2023 (N=260; 1820 choice observations)

*Attribute*	*β*	*SE*	*z*	*p*	*95% CI*	*Rel. importance (%)*	*Preferred level*
**Organizer**	0.0358	0.0420	0.85	0.394	–0.0465–0.1181	6.0	
**Content format**	-0.1899***	0.0421	-4.51	<0.001	-0.2723 – -0.1074	32.0	Video
**Organization mode**	-0.0094	0.0420	-0.22	0.822	-0.0917–0.0728	1.6	
**Frequency**	0.0569	0.0421	1.35	0.176	-0.0256–0.1394	9.6	
**Educational content**	-0.1320**	0.0422	-3.13	0.002	-0.2147 – -0.0493	22.2	Cigarette + e-cigarette
**Delivery location**	-0.1698***	0.0422	-4.03	<0.001	-0.2525 – -0.0872	28.6	Online

Effects coding (+1/-1). Log-likelihood= -1198.34. Relative importance = |β_i| / Σ|β| × 100. DCE: discrete choice experiment. SE: standard error.

* p<0.05,

** p<0.01,

*** p<0.001.

**Figure 1:**
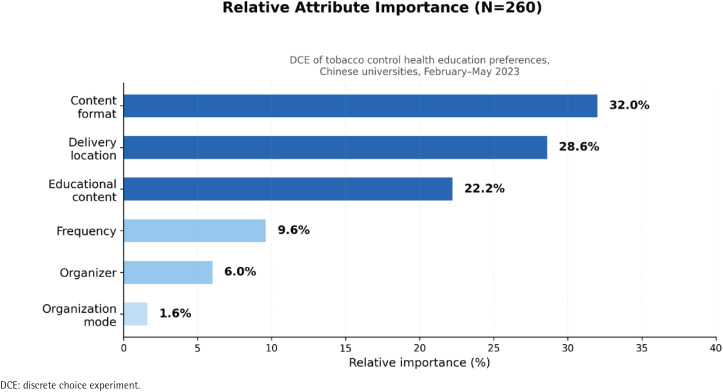
Relative attribute importance in the full-sample conditional logit model of a DCE of tobacco control health education preferences among Chinese university students, February–May 2023 (N=260)

The remaining three attributes – organizer (β=0.0358; 95% CI: -0.0465–0.1181, p=0.394), frequency (β=0.0569; 95% CI: -0.0256–0.1394, p=0.176), and organization mode (β= -0.0094; 95% CI: -0.0917–0.0728, p=0.822) – did not reach statistical significance. Relative attribute importance analysis (Figure 1) revealed that content format (32.0%), delivery location (28.6%), and educational content (22.2%) together accounted for 82.8% of total preference weight, while organizer (6.0%), frequency (9.6%), and organization mode (1.6%) together contributed only 17.2%.

### Exploratory subgroup analyses

**Figure 2:**
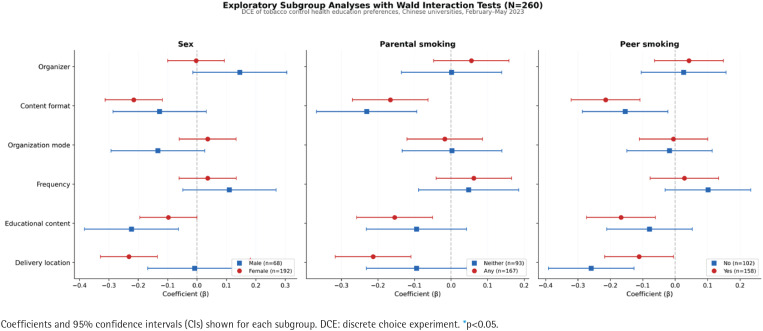
Forest plot of exploratory subgroup analyses with Wald interaction tests in a DCE of tobacco control health education preferences among Chinese university students, February–May 2023 (N=260)

Stratified conditional logit models and Wald interaction tests were conducted to explore preference heterogeneity by sex, parental smoking status, and peer smoking status (Table 5) (Figure 2).

**Table 5:** Exploratory subgroup analyses by sex, parental smoking (PS), and peer smoking (FS) in a DCE of tobacco control health education preferences, Chinese universities, February–May 2023 (N=260)

*Attribute*	*Male β (95% CI)*	*Female β (95% CI)*	*p_int*	*No PS β (95% CI)*	*PS β (95% CI)*	*p_int*	*No FS β (95% CI)*	*FS β (95% CI)*	*p_int*
**Total**, n	68	192		93	167		102	158	
**Organizer**	0.1459 (-0.01–0.31)	-0.0032 (-0.10–0.09)	0.118	0.0014 (-0.14–0.14)	0.0556 (-0.05–0.16)	0.535	0.0263 (-0.10–0.16)	0.0424 (-0.06–0.15)	0.851
**Content format**	-0.1274 (-0.29–0.03)	-0.2153 (-0.31 – -0.12)	0.355	-0.2308 (-0.37 – -0.09)	-0.1661 (-0.27 – -0.06)	0.460	-0.1543 (-0.29 – -0.02)	-0.2145 (-0.32 – -0.11)	0.485
**Organization mode**	-0.1333 (-0.29–0.03)	0.0362 (-0.06–0.13)	0.075	0.0028 (-0.13–0.14)	-0.0171 (-0.12–0.09)	0.820	-0.0177 (-0.15–0.11)	-0.0052 (-0.11–0.10)	0.885
**Frequency**	0.1099 (-0.05–0.27)	0.0363 (-0.06–0.13)	0.438	0.0483 (-0.09–0.19)	0.0624 (-0.04–0.17)	0.872	0.1013 (-0.03–0.23)	0.0283 (-0.08–0.13)	0.398
**Educational content**	-0.2229 (-0.38 – -0.06)	-0.0975 (-0.19–0.00)	0.189	-0.0945 (-0.23–0.04)	-0.1542 (-0.26 – -0.05)	0.497	-0.0791 (-0.21–0.05)	-0.1672 (-0.27 – -0.06)	0.309
**Delivery location**	-0.0077 (-0.17–0.15)	-0.2319 (-0.33 – -0.13)	0.019*	-0.0945 (-0.23–0.04)	-0.2131 (-0.32 – -0.11)	0.176	-0.2590 (-0.39 – -0.13)	-0.1112 (-0.22–0.00)	0.087

Effects coding (+1/-1). p_int: Wald interaction test p-value. PS: parental smoking. FS: peer smoking. DCE: discrete choice experiment.

* p<0.05.

In the sex-stratified analysis (68 males vs 192 females), a statistically significant interaction was detected for delivery location (Wald p=0.019): female students showed a preference for online delivery (β= -0.2319; 95% CI: -0.3291 – -0.1346), whereas male students showed near-indifference (β= -0.0077; 95% CI: -0.1680–0.1526). No other sex-based interaction reached statistical significance (all p>0.07).

In the parental smoking–stratified analysis (93 with neither parent smoking vs 167 with at least one parent smoking), no significant interactions were detected (all p>0.18).

In the peer smoking–stratified analysis (102 with no close friends smoking vs 158 with at least one close friend smoking), no interactions reached the p<0.05 threshold, though the delivery location interaction approached significance (Wald p=0.087). Students without smoking peers showed a stronger preference for online delivery (β= -0.2590; 95% CI: -0.3909 – -0.1271) compared with those whose peers smoke (β= -0.1112; 95% CI: -0.2175 – -0.0048).

## DISCUSSION

This study applied DCE methodology to quantify Chinese never-tobacco-using university students’ preferences for tobacco control health education program attributes – a topic that has received limited attention in the existing DCE literature. By embedding the DCE within a PMT-based RCT, we were able to simultaneously obtain preference estimates and empirically test whether intervention exposure influenced stated preferences.

### Content format: The primacy of video-based delivery

The finding that content format was the most important attribute, with students preferring video-based over text/image formats, is consistent with evidence on the effectiveness of video-based health education^29,30^. The strong preference for video content aligns with the media consumption patterns of contemporary Chinese university students, who are immersed in short-video platforms such as Douyin (TikTok) and Bilibili. Recent research has demonstrated the effectiveness of Bilibili as a platform for youth-oriented smoking cessation health promotion^31^, while a DCE study by Xiao et al.^32^ examining preferences for health science popularization short videos in China, found that the Chinese public, including young adults, strongly preferred video-based health content delivered through accessible digital formats. These findings collectively suggest that tobacco control programs relying primarily on printed materials or static presentations may face engagement challenges with this population.

### Delivery location: The preference for online education

Delivery location emerged as the second most influential attribute, with students preferring online over offline formats. During the COVID-19 pandemic, over 1450 Chinese colleges conducted online education, reaching 17.75 million university students^33^. A systematic review of health sciences students’ online learning experiences found that flexibility was the most frequently cited advantage of online delivery^34^. The preference for online delivery may also reflect the scheduling convenience that online formats afford.

### Educational content: Demand for comprehensive coverage

Students’ preference for comprehensive content covering both cigarette and e-cigarette harms suggests that students value an integrated approach to tobacco education. This result aligns with the recommendations of Fang et al.^35^ who reported knowledge gaps about e-cigarettes among Chinese university students and advocated for incorporating e-cigarette content into campus health education.

### Non-significant attributes

The non-significance of organizer (teacher/expert vs student-led), organization mode (teaching-led vs self-study), and frequency (>1 vs 1 per semester) is noteworthy. The indifference between organizer types contrasts with the study of Choi and Templin^17^ who reported that US college students valued two-way communication in tobacco treatment settings. The discrepancy may reflect cultural differences in educational expectations between Chinese and Western university environments. The non-significance of frequency suggests that students prioritize how education is delivered over how often it occurs.

### Validation of the embedded DCE–RCT design

A methodological contribution of this study is the empirical demonstration that PMT intervention exposure did not confound DCE preference measurement. All six Wald interaction tests comparing intervention and control arms were non-significant (all p>0.32). This supports the conclusion that the embedded design did not introduce systematic bias. The ISPOR task force has noted the importance of testing for context effects in DCEs^23,24^; our embedded design provided a natural experiment for this purpose.

### Preference heterogeneity

The significant sex × delivery location interaction (p=0.019) provides preliminary evidence that the overall preference for online delivery was driven primarily by female students. The sex ratio in our sample (73.8% female) is broadly consistent with enrollment patterns in Chinese universities. A purely online format may not optimally engage male students, and blended delivery options could be considered. The near-significant peer smoking × delivery location interaction (p=0.087) tentatively suggests that students whose close friends smoke may be less averse to in-person education. These exploratory findings should be interpreted with caution and require confirmation in independent, adequately powered studies, as they were not pre-specified and involve multiple comparisons.

### Strengths and limitations

-Study strengths include the application of DCE methodology to an understudied domain, the embedded RCT design permitting validation, a sample exceeding published recommendations^27,28^, and reporting in accordance with the DIRECT checklist^25^.

Several limitations should be acknowledged. First, participants were recruited through online convenience sampling, and the sample was predominantly female and enrolled in undergraduate programs, which may limit generalizability. Second, the binary forced-choice format without an opt-out may overstate the strength of preferences. Third, the fixed presentation order of choice sets could introduce order effects. Fourth, the restriction to never-tobacco-users means findings may not generalize to current users. Fifth, the conditional logit model assumes preference homogeneity; future studies could employ mixed logit or latent class models. Sixth, as all data were self-reported, recall bias and social desirability bias cannot be excluded. Seventh, the study did not include a dominant alternative test or repeated choice set for test-retest reliability. Finally, findings are derived from Chinese university students and may reflect cultural norms and educational practices specific to China, which may limit generalizability to other contexts.

## CONCLUSIONS

Chinese university students who have never used tobacco prefer tobacco control health education programs that employ video-based content, are delivered online, and comprehensively address both cigarette and e-cigarette harms. These three attributes account for approximately 83% of total preference weight. The absence of preference differences between intervention and control arms supports the validity of the embedded DCE–RCT methodology. These findings provide preliminary demand-side evidence that may inform the design of future campus-based prevention programs targeting never-tobacco-using students. Further research with broader populations – including current tobacco users – is needed before definitive program design recommendations can be made.

## Supplementary Material



## Data Availability

The data supporting this research are available from the authors on reasonable request.
