# Supplementary material for: Preferences for tobacco control health education among Chinese university students: A discrete choice experiment embedded in a randomized controlled trial
Source: Tob Induc Dis. 2026 Jul 1;24:10.18332/tid/220332. doi: 10.18332/tid/220332 (PMC13332694; doi:10.18332/tid/220332)
Supplement: Supplementary file 1 [file TID-24-102-s1.pdf]

# Supplementary Table S1. DIRECT Checklist for Discrete Choice Experiments in Health

Reference: Ride J, Goranitis I, Meng Y, LaBond C, Lancsar E. PharmacoEconomics. 2024;42(10):1161–1175. doi:10.1007/s40273-024-01431-6

Compliance summary: 20 of 26 items fully reported | 4 partially reported | 2 not reported

| Domain | Domain name | # | Criterion | Status | Location / comment |
|---|---|---|---|---|---|
| 1 | Purpose & rationale | 1 | Describe the real-world context and decision-maker. | **Yes** | Introduction, paragraphs 1–4. Informs tobacco control education program design. |
| 1 | | 2 | Provide rationale for using a DCE. | **Yes** | Introduction, paragraph 3. DCE for quantitative attribute importance estimation. |
| 2 | Attributes & levels | 3 | Describe how attributes and levels were derived. | **Yes** | Methods: DCE Design. Literature review, expert consultation, student focus groups. |
| 2 | | 4 | Provide the final list of attributes and levels. | **Yes** | Table 1. Six attributes with two levels each. |
| 3 | Experimental design | 5 | Report number of alternatives per choice set; labelled or unlabelled. | **Yes** | Methods: DCE Design. Two unlabelled alternatives per choice set. |
| 3 | | 6 | Describe response options. | **Yes** | Binary forced choice with no opt-out. |
| 3 | | 7 | Describe type of experimental design. | **Yes** | Main-effects fractional factorial with near-orthogonality (r=−0.17). |
| 3 | | 8 | Describe which effects are identified. | **Yes** | Methods: Statistical Analysis. Main effects only. |
| 3 | | 9 | Report number of choice sets, blocks, choice sets per block. | **Yes** | 7 choice sets; no blocking; all respondents completed all 7. |
| 3 | | 10 | Indicate how experimental design was obtained. | **Yes** | Main-effects fractional factorial; specific software not reported. |
| 4 | Survey design | 11 | Provide sample choice set and instructions. | **Yes** | Table 1 provides attribute/level definitions. Full instrument available on request. |
| 4 | | 12 | Report any randomisation of order. | **Yes** | Fixed order. Acknowledged as limitation. |
| 4 | | 13 | Describe what was checked in piloting. | **Yes** | Pilot with focus group participants; details in parent RCT protocol. |
| 4 | | 14 | Report whether pilot information updated the design. | **Yes** | Not reported. |
| 5 | Sample & data collection | 15 | Report inclusion/exclusion criteria. | **Yes** | Methods: Participants. Age 18–24, full-time, never-tobacco-users. |
| 5 | | 16 | Describe how data were collected. | **Yes** | Online self-administered via WenJuanXing at week 4 follow-up. |
| 5 | | 17 | Report response/cooperation rate. | **Yes** | 260/289 = 89.97% valid response rate. |
| 5 | | 18 | Report final sample size and how determined. | **Yes** | N=260. Determined by parent RCT enrollment |
| 5 | | 19 | Describe respondent characteristics. | **Yes** | Table 2. Sex, education, alcohol, exercise, parental/peer smoking, trial arm. |
| 6 | Econometric analysis | 20 | Indicate coding of data. | **Yes** | Effects coding (+1/−1); reference levels defined in Table 1. |
| 6 | | 21 | Report whether any respondents were removed. | **Yes** | 29 of 289 RCT participants did not complete DCE; 260 valid responses analyzed. |
| 6 | | 22 | Provide rationale for model choice. | **Yes** | Conditional logit; random utility theory; IIA assumption. |
| 6 | | 23 | Report model specification. | **Yes** | Utility function with 6 main-effect terms; Methods section. |
| 7 | Reporting of results | 24 | Report model performance. | **Yes** | Pseudo-$R^2$=0.050 reported in Table 4. |
| 7 | | 25 | Describe methods for analysis of results. | **Yes** | Relative importance; stratified models; Wald interaction tests. |
| 7 | | 26 | Report measures of precision. | **Yes** | Tables 3–5: 95% CIs, SEs, and p-values for all coefficients. |

Color key: White = fully reported; Yellow = partially reported; Red = not reported.

© **2026 Chen Y. et al.**
